# Onset of human preterm and term birth is related to unique inflammatory transcriptome profiles at the maternal fetal interface

**DOI:** 10.7717/peerj.3685

**Published:** 2017-09-01

**Authors:** Radek Bukowski, Yoel Sadovsky, Hani Goodarzi, Heping Zhang, Joseph R. Biggio, Michael Varner, Samuel Parry, Feifei Xiao, Sean M. Esplin, William Andrews, George R. Saade, John V. Ilekis, Uma M. Reddy, Donald A. Baldwin

**Affiliations:** 1 Dell Medical School, Department of Women’s Health, University of Texas at Austin, Austin, TX, United States of America; 2 Magee-Womens Research Institute, University of Pittsburgh, Pittsburgh, PA, United States of America; 3 Department of Biophysics & Biochemistry, University of California, San Francisco, San Francisco, CA, United States of America; 4 School of Public Health, Department of Biostatistics, Yale University, New Haven, CT, United States of America; 5 School of Medicine, Department of Obstetrics and Gynecology, University of Alabama - Birmingham, Birmingham, AL, United States of America; 6 School of Medicine, Intermountain Healthcare, Department of Obstetrics and Gynecology, University of Utah, Salt Lake City, UT, United States of America; 7 School of Medicine, Department of Obstetrics and Gynecology, University of Pennsylvania, Philadelphia, PA, United States of America; 8 Arnold School of Public Health, Department of Epidemiology and Biostatistics, University of South Carolina, Columbia, SC, United States of America; 9 Department of Obstetrics and Gynecology, University of Texas Medical Branch at Galveston, Galveston, TX, United States of America; 10 Pregnancy and Perinatology Branch, Eunice Kennedy Shriver National Institute of Child Health and Human Development, Bethesda, MD, United States of America; 11 Signal Biology Inc., Philadelphia, PA, United States of America

**Keywords:** Pregnancy, Pretrem labor, Labor, Gene expression, Transcriptomics

## Abstract

**Background:**

Preterm birth is a main determinant of neonatal mortality and morbidity and a major contributor to the overall mortality and burden of disease. However, research of the preterm birth is hindered by the imprecise definition of the clinical phenotype and complexity of the molecular phenotype due to multiple pregnancy tissue types and molecular processes that may contribute to the preterm birth. Here we comprehensively evaluate the mRNA transcriptome that characterizes preterm and term labor in tissues comprising the pregnancy using precisely phenotyped samples. The four complementary phenotypes together provide comprehensive insight into preterm and term parturition.

**Methods:**

Samples of maternal blood, chorion, amnion, placenta, decidua, fetal blood, and myometrium from the uterine fundus and lower segment (*n* = 183) were obtained during cesarean delivery from women with four complementary phenotypes: delivering preterm with (PL) and without labor (PNL), term with (TL) and without labor (TNL). Enrolled were 35 pregnant women with four precisely and prospectively defined phenotypes: PL (*n* = 8), PNL (*n* = 10), TL (*n* = 7) and TNL (*n* = 10). Gene expression data were analyzed using shrunken centroid analysis to identify a minimal set of genes that uniquely characterizes each of the four phenotypes. Expression profiles of 73 genes and non-coding RNA sequences uniquely identified each of the four phenotypes. The shrunken centroid analysis and 10 times 10-fold cross-validation was also used to minimize false positive finings and overfitting. Identified were the pathways and molecular processes associated with and the cis-regulatory elements in gene’s 5′ promoter or 3′-UTR regions of the set of genes which expression uniquely characterized the four phenotypes.

**Results:**

The largest differences in gene expression among the four groups occurred at maternal fetal interface in decidua, chorion and amnion. The gene expression profiles showed suppression of chemokines expression in TNL, withdrawal of this suppression in TL, activation of multiple pathways of inflammation in PL, and an immune rejection profile in PNL. The genes constituting expression signatures showed over-representation of three putative regulatory elements in their 5′and 3′ UTR regions.

**Conclusions:**

The results suggest that pregnancy is maintained by downregulation of chemokines at the maternal-fetal interface. Withdrawal of this downregulation results in the term birth and its overriding by the activation of multiple pathways of the immune system in the preterm birth. Complications of the pregnancy associated with impairment of placental function, which necessitated premature delivery of the fetus in the absence of labor, show gene expression patterns associated with immune rejection.

## Introduction

Preterm birth is a main determinant of neonatal mortality and morbidity, and a major contributor to overall mortality and burden of disease (Lopez et al., 2006; Mathers & Loncar, 2006). However, its molecular underpinnings remain elusive (Muglia & Katz, 2010). This is mainly the result of complex molecular phenotype with many molecular processes and tissue types underlying parturition, as well as difficulties in precise clinical phenotyping of the disease (Muglia & Katz, 2010; Romero, Dey & Fisher, 2014; Smith, 2007). Phenotyping of the preterm birth is especially difficult when delivery occurs by cesarean section, which is the mode of delivery in almost half of the early preterm births (Bettegowda et al., 2008). These challenges are further confounded by overlap of molecular processes associated with labor and with physiological changes occurring with advancing pregnancy.

Thus, parturition can be generally viewed as four complementary phenotypes, based on the timing and presence of labor: delivery in women at term with labor (TL), delivery at term without labor (TNL), delivery after preterm labor (PL) and preterm delivery without labor (PNL), due to fetal or maternal indications. However, these phenotypes share molecular processes related to labor itself or to changes associated with advancement of pregnancy. The molecular processes related to labor and present in both PL and TL are associated with common final pathways of labor related to uterine contractions, cervical ripening and fetal membrane rupture (Romero, Dey & Fisher, 2014). Identification of a unique gene expression profile of each of the four phenotypes and differentiating one phenotype from the remaining three will exclude from the analysis molecular processes common to two or more groups. In this analysis gene expression profiles related to the common final pathway of labor and common to PL and TL will be excluded from the analysis, and excluding them will unveil processes preceding these final changes.

The molecular processes underlying preterm and term labor occur simultaneously in multiple tissues composing the pregnancy and located within the uterus (Romero, Dey & Fisher, 2014; Smith, 2007). The proximity of these tissues and their molecular interactions integrate them into a functionally single unit, of a pregnant uterus in which concerted molecular processes result in preterm or term birth (Norwitz, Robinson & Challis, 1999; Racicot et al., 2014; Romero, Dey & Fisher, 2014; Smith, 2007).

The multiplicity of genes, proteins, signals and processes involved, coupled with our limited understanding of the processes underlying parturition suggests a whole-genome high-throughput approach as the most suitable strategy to investigate molecular underpinnings of preterm birth. However, such an approach, due to the high ratio of genes to samples and to outcomes, is also associated with high risk of severe overfitting. In overfitted analyses the samples under study are well described, but inferences may not be representative of other samples or the population in general. Thus, overfitting results in low generalizability or external validity, and a high false positive rate. Strategies shown to effectively decrease the effect of overfitting include “shrinkage” of estimates (Steyerberg et al., 2001) and cross-validation (Shi et al., 2010). To comprehensively evaluate the molecular processes underlying preterm and term labor while accounting for the difficulties associated with the study of preterm birth, we profiled the transcriptome of the tissues comprising the pregnant uterus using techniques to minimize overfitting among four very precisely phenotyped groups of women: preterm and term, with or without labor. This analysis was performed as a part of the *Eunice Kennedy Shriver* National Institute of Child Health and Human Development (NICHD) funded Genomics and Proteomics Network for Preterm Birth Research.

## Materials and Methods

### Ethics statement

The study was approved by the Institutional Review Boards of University of Alabama at Birmingham, University of Texas Medical Branch at Galveston, University of Utah and Yale University. IRB# 06-355, 06-356, 06-357. All mothers participating in the study gave written informed consent.

### Study design

The NICHD Genomic and Proteomic Network (GPN) for Preterm Birth Research is composed of three primary clinical sites (University of Alabama at Birmingham, University of Texas Medical Branch at Galveston, and University of Utah), a laboratory core (University of Pennsylvania), a data management, statistics, and informatics core (Yale University), NICHD, and a steering committee chair. We profiled the transcriptome of the tissues comprising the pregnant uterus among four very precisely phenotyped groups of women: preterm and term, with or without labor.

### Phenotype definition

Patients admitted prior to delivery whose gestational age at admission was between 24 weeks 0 days and 34 weeks 0 days or between 39 weeks 0 days and 41 weeks 6 days were eligible for the study. Because this study required the collection of multiple uterine tissues specimens at the time of delivery, only patients delivering by cesarean delivery were eligible.

Spontaneous onset of labor was defined as ≥4 spontaneously occurring contractions (each >30 sec duration) in a 20 min period of time or 10 contractions per hour **and** absolute dilation of ≥2 cm **and** one of the following: increase in dilation of ≥1 cm **or** effacement of ≥75%. Preterm premature rupture of the membranes (PPROM) was defined as evidence of ruptured membranes between 20 weeks and 0 days and 34 weeks 0 days that precedes the onset of spontaneous regular contractions within 24 h.

We identified four phenotypes: PL was defined as delivery at 24 weeks 0 days to 34 weeks 0 days with spontaneous onset of labor, defined above, with or without PPROM. PNL was a delivery at 24 weeks 0 days to 34 weeks 0 days without evidence of labor with or without PPROM that was not followed by the onset of spontaneous regular contractions, but instead was managed at least 24 h later with elective delivery. TL was defined as delivery between 39 weeks 0 days and 41 weeks and 6 days with spontaneous onset of labor and without rupture of membranes before onset of labor. TNL phenotype was a delivery between 39 weeks 0 days and 41 weeks and 6 days without spontaneous, induced or augmented labor and without rupture of membranes before the onset of labor. The exclusion criteria for all the phenotypres were: maternal uterine anomalies, multi-fetal gestation, known aneuploidy or lethal fetal anomalies, polyhydramnios, serious maternal medical conditions and cervical cerclage.

Enrolled were 35 pregnant women in 4 precisely defined phenotypes: PL (*n* = 8), PNL (*n* = 10), TL (*n* = 7) and TNL (*n* = 10). The indications for PNL delivery were: preeclampsia (3), fetal non-reassuring status (4), bleeding related to placenta previa (1) and placental abruption (1), and preterm premature rupture of membranes with intrauterine infection and without evidence of labor (1). Five of the ten PNL pregnancies had additionally evidence of intrauterine growth restriction and none had evidence of labor according to study criteria. All participants gave written informed consent, and the study was approved by the Institutional Review Boards of all participating institutions.

### Tissues

Samples of maternal blood, chorion, amnion, placenta, decidua, fetal blood, and myometrium from the uterine fundus and lower segment (*n* = 183) were obtained during cesarean delivery, placed on ice in sterile containers, and processed immediately in a clean area. Maternal and fetal cord blood samples were collected using the PAXgene Blood RNA system (PreAnalytiX/Qiagen, Valencia CA, USA). Placental samples were processed according to a previously published protocol (Burton et al., 2014). Chorion, amnion, decidua, uterine fundus and lower segment samples were rinsed in cold PBS, placed in TRIzol Reagent (Life Technologies, Grand Island NY, USA) and flash frozen in liquid nitrogen. All samples were shipped in dry ice and stored at −80 °C.

### RNA extraction

Maternal and fetal cord blood samples collected in PAXgene Blood RNA tubes were processed according to the vendor’s protocol (PreAnalytiX/Qiagen, Valencia CA, USA) for purification of total RNA. Tissue samples, collected in TRIzol, were homogenized before phase separation with chloroform, and RNA in the aqueous phase was purified using RNeasy spin columns (Qiagen, Valencia, CA, USA). RNA quality and quantity were assessed by spectrophotometry and on a Bioanalyzer 2100 with RNA 6000 Nano LabChips (Agilent Technologies, Santa Clara CA, USA).

### Microarray analysis

The analyses were conducted in the analytic center using the vendors’ recommended methods. The Ovation Pico WTA System v2 (NuGEN Technologies, San Carlos CA, USA) was used with 50 ng of input total RNA, providing linear expansion of all transcripts without interference from ribosomal or globin RNAs. The resulting cDNA was labeled with biotin (Encore Biotin Module; NuGEN) and hybridized to Human Gene 1.1 ST Array Plates (Affymetrix, Santa Clara CA, USA) followed by scanning on an Affymetrix GeneTitan instrument and inspection of quality control metrics and probeset summarization with Expression Console software. Microarray data sets were analyzed using Partek Genomics Suite (Partek, St. Louis MO, USA) with normalization by the RMA algorithm (Irizarry et al., 2003).

### Quantitative PCR

PCR validation was performed in 23 chorion and 21 amnion samples. Since it was not feasible to validate all the samples, the PCR validation was performed on the tissues with the largest variation in gene expression among the phenotypes, thus the ones contributing the most to the unique gene expression profiles characterizing the phenotypes. To internally validate the results of the microarray analyses the samples from the same patients were analyzed using the PCR. We have selected four genes for the PCR validations, which have been previously reported to be involved in preterm birth. They were not selected from a larger group of genes. Each measurement of relative abundance of four genes in each of the four phenotypes was performed in duplicate. Reverse transcription was performed on 400 ng total RNA using High-Capacity RNA-to-cDNA Kit (Cat # 4387406; Life Technologies, Grand Island, NY, USA). qPCR was performed in duplicate, using SYBR Select enzyme master mix (Cat # 4472920; Life Technologies) and the ViiA7 Real-Time PCR System (Life Technologies). The primer sequences used in this study are detailed in Table S5, and all primers were synthesized by Integrated DNA Technologies (IDT, Coralville, IA, USA). Transcript expression level was normalized to the expression level of GAPDH. The fold change, relative to control samples, was determined by the 2^−ΔΔCt^ method (Livak & Schmittgen, 2001).

### Statistical analysis

#### Gene expression profiles

The different samples of tissues were treated as independent samples, and each contributed an expression profile rather than being averaged over all tissues or within tissue type. Cumulatively those individual samples expression profiles provide expression profile of pregnancy in preterm and term pregnancies in labor and the absence of labor.

To identify tissues with the largest differences in gene expression in each tissue a transformation was performed followed by calculation of a proportion of genes with the largest gene expression variation:

(1) *z*-score normalization of the full dataset (all 183 samples normalized together) to obtain the normalized quantities
  \begin{eqnarray*}z[g,i]=(x[g,i]-\mathrm{mu}[g])/\mathrm{sigma}[g] \end{eqnarray*}
  for each gene  *g* and sample  *i*, where mu[*g*] is the average over all samples  *i* of  *x*[*g*, *i*] and sigma[*g*] is the standard deviation of *x*[*g*, *i*] over all samples *i* followed by
(2) calculating within-tissue standard deviations  *s*[*g*, *t*] of these *z*-scores for each gene  *g* and tissue  *t*:
  \begin{eqnarray*}s[g,t]=\mathrm{stddev}(\{z[g,i]:i\text{is a sample from tissue}t\}) \end{eqnarray*}
(3) for each tissue *t*, applying a filter to retain only genes  *g* for which  *s*[*g*, *t*] > 1 or  *s*[*g*, *t*] > 2.

Since the value  *s*[*g*, *t*] obtained for a given gene  *g* in tissue  *t* is thus defined in terms of multiples of sigma [*g*], the standard deviation of the expression values of g over the full dataset, but  *s*[*g*, *t*] is not itself a *z*-score; thus there is no reason to expect that the quantities  *s*[*g*, *t*] will follow a standard normal distribution with corresponding proportions of genes above the one or two standard deviations.

The normalized gene expression data were analyzed using shrunken centroid approach (Tibshirani et al., 2002). This technique identifies a minimal set of genes that characterizes each of the four phenotypes. In this approach, class centroids are calculated as average expression of each gene in each of the four phenotypes. Next, for the gene expression profile of each sample, a squared distance to each of the four phenotype centroids is calculated. The predicted phenotype is the one whose centroid is closest to the expression profile of the sample. To decrease the effect of overfitting, the phenotype centroids are shrunk towards the overall centroid (average gene expression for all the four phenotypes) after standardizing by the within-phenotype standard deviation for each gene. The standardization gives larger weight to genes with stable expression within the same phenotype. The amount of shrinkage was determined by 10 times 10-fold cross-validation. The cross-validation used to determine the amount of shrinkage was separate and was not nested within the cross-validation used to determine the classification performance.

#### Cross validation

The 10 times 10-fold cross-validation was also used to determine the accuracy of the gene sets identified using shrunken centroid analysis in ascertainment of the four phenotypes (Shi et al., 2010). In this process, the samples were randomly divided into 10 approximately equal size parts. The 10 parts were balanced regarding proportion of each phenotypic group. The model was developed in a set that comprised nine parts, and then phenotypes were predicted in the remaining one part, serving as a test sample. This procedure was repeated 10 times, with each part playing the role of a test sample. The errors of each part were then used to calculate the overall accuracy in classification of the four phenotypes.

#### Gene pathways

The pathways and molecular processes impacted by genes expressions associated with the four phenotypes were identified using iPAGE pathway analysis (Goodarzi, Elemento & Tavazoie, 2009). This information-theoretic pathway analysis method directly quantifies mutual information between pathways and expression profiles. Mutual information is a measure of dependence between variables, in this case between expression profiles of the four phenotypes and Gene Ontology or MSigDB pathways (Ashburner et al., 2000; Subramanian et al., 2005). The mutual information was calculated as the relative entropy between the joint distribution and the product distribution of gene expression and known pathways in the Gene Ontology. *Z*-score associated with the MI value was calculated using 10,000 randomization tests. Background gene expression pattern was obtained from published gene expression experiments performed in different tissues (Goodarzi, Elemento & Tavazoie, 2009). Nonparametric statistical tests were then used to determine whether a pathway is significantly informative about the expression profile. Enrichment and depletion of pathway components across the four phenotypes contribute to the mutual information. The method incorporates the conditional mutual information only to pathways, which are independently informative about the expression profiles of the four phenotypes.

#### Regulatory elements

The *cis*-regulatory elements in gene’s 5′ promoter or 3′-UTR regions, which best explain RNA expression profiles in the four phenotypes, were identified using FIRE analysis (Elemento, Slonim & Tavazoie, 2007). This analysis uses mutual information to determine whether a 9-mer motif’s presence or absence in the 5′-or 3′-UTR regulatory regions of a given gene provides significant information regarding the expression of that gene in each of the four phenotypes. Candidate seeds consist of the entire set of 7-mers, and the motifs are set to be nine nucleotides long. The regulators responsible for the observed expression profiles in the four phenotypes were identified by comparing the putative regulatory elements to transcription factor binding sites in JASPAR (Sandelin et al., 2004) and TRANSFAC (Matys et al., 2006) databases and to seed regions of known miRNAs (Griffiths-Jones et al., 2008).

#### Regulatory elements associated with the pathways

The regulatory elements identified using FIRE analysis were associated with pathways identified by iPAGE. This association reveals potential mechanisms by which the pathways are regulated through the putative binding sites for regulatory proteins or miRNAs. This strategy again quantifies the extent to which independently identified pathways and cis-regulatory elements are mutually informative (Goodarzi, Elemento & Tavazoie, 2009). Mutual information quantifies the relationships between pathways and cis-regulatory elements. Non-parametric statistical tests are then used to determine if the putative regulatory element is informative about the deregulated pathway (Goodarzi, Elemento & Tavazoie, 2009).

## Results

### Transcriptional signature

We identified 73 genes and non-coding RNA sequences in tissues comprising pregnancy with expression profiles that uniquely identified the four phenotypes. Among those genes, 50 were associated with PL, six with PNL, six with TL and 23 with TNL. The expression of some of the genes was associated with more than one phenotype. The 50 genes associated with PL were all expressed higher in PL than in the remaining three phenotypes except for SCARNA17, P2RY14, CXCL9 and PDK4 (Table S1). The six genes that identified PNL were all expressed higher in PNL than in other phenotypes (Table S2). Also, genes identifying TL were all expressed higher in TL except for mitochondrially encoded tRNA glycine (MT-TG) (Table S3). All genes identifying TNL were expressed lower in the TNL than in the remaining three phenotypes (Table S4).

These transcription signatures correctly classified 42% of the samples into one of the four phenotypes using 10 times 10-fold cross-validation (Table 1). The accuracy of classification varied substantially among the four phenotypes, from 10.5% correctly classified samples of TL, 16.4% of PNL, 47.1% of PL to 85.7% of TNL samples. The classification accuracy did not correlate with the number of samples in each phenotype. In comparison, a random classifier, in the absence of any information from the expression profiles would on average correctly classify 25% of the phenotypes or samples. However, when the expression profiles provide information about the phenotypes some phenotypes will be classified with better and some with worse accuracy than expected on average at random. This is due to differences in underlying biological processes, especially gene expression, and in sizes of phenotypic groups.

**Table 1:** Expression profiles of the four phenotypes. Expression profile of the set of genes associated with a phenotype. Phenotype of samples ((preterm labor (P L); Preterm non-labor (P NL); Term labor (T L); Term non-labor (T NL)). Number and percent of samples of particular phenotype identified by each of the expression profiles associated with a particular phenotype.

Expression profile	Phenotype			
	P L (*n* = 34)	P NL (*n* = 55)	T L (*n* = 38)	T NL (*n* = 56)
P L	**16 (47%)**	11 (20%)	8 (21%)	3 (5%)
P NL	2 (6%)	**9 (16%)**	7 (18%)	4 (7%)
T L	1 (3%)	1 (2%)	**4 (11%)**	1 (2%)
T NL	15 (44%)	34 (62%)	19 (50%)	**48 (86%)**

The permutation testing has shown that applying 10x-repeated 10-fold cross-validation using an inner 10-fold nested cross-validation to optimize the shrinkage parameter to the training set with the class labels randomly permuted, to disrupt any true relationship between class and expression values, produces consistently lower accuracy estimates than observed in the study. Randomly generated 100 permutations, provided 100 accuracy estimates ranging from 23.88% to 37.70%, with a mean of 28.71% and a standard deviation of 2.61%. Since all 100 permutations resulted in accuracy estimates below that observed using the actual phenotype labels, we can reject with 95% confidence, the null hypothesis that the classification using real phenotype labels is no better than could be expected by chance, the exact permutation test *p*-value <0.036 (using the Pearson-Klopper method for estimating binomial probability confidence intervals).

**Figure 1:**
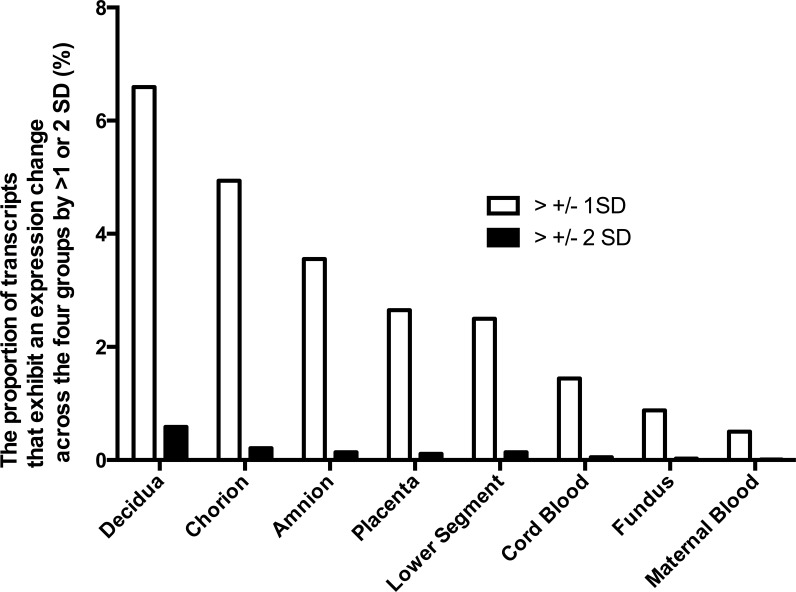
Gene expression differences across the phenotypes in tissues of the pregnant uterus. The proportion of genes which expression across the four phenotypes (term non-labor (TNL), preterm non-labor (PNL), term labor (TL) and preterm labor (PL)) differs more than ±1 or ±2 standard deviations (SD).

### Tissue expression profile

The differences in gene expression among the four phenotypes varied also among the tissues. Characteristics of gene expression in each tissue are shown to identify tissues where the largest differences in gene expression occur across the phenotypes. To identify tissues with the largest differences in gene expression, the expression of each gene in each sample from a given tissue across the four phenotypes was calculated. Proportion of genes with expression variability across the four phenotypes larger than one or two SD was shown. However, to obtain expression profile of pregnancy in preterm and term pregnancies in labor and the absence of labor, different samples of tissues were treated as independent samples, and each contributed an expression profile. The largest differences in gene expression among the four phenotypes were observed in decidua, chorion and amnion (Fig. 1). In those tissues, the proportion of genes with expression differences more than one or two standard deviations (SD) among the phenotypes was several times larger than in the fundus, maternal blood and fetal blood. This observation indicates that gene expression differences among the four phenotypes were highest in the decidua, amnion and chorion rather than in the fundus and lower segment of the uterus or in the maternal or fetal blood. An analysis including four tissues with the largest gene expression variability across the phenotypes (decidua, chorion, amnion, and placenta) showed that gene expression profile of this subset of tissues classified the four phenotypes worse than the full set of tissues. The gene expression profiles of the subset of tissues correctly classified 75% of P L, 96% of T NL, but only 7% of P NL and 0% of T L.

**Figure 2:**
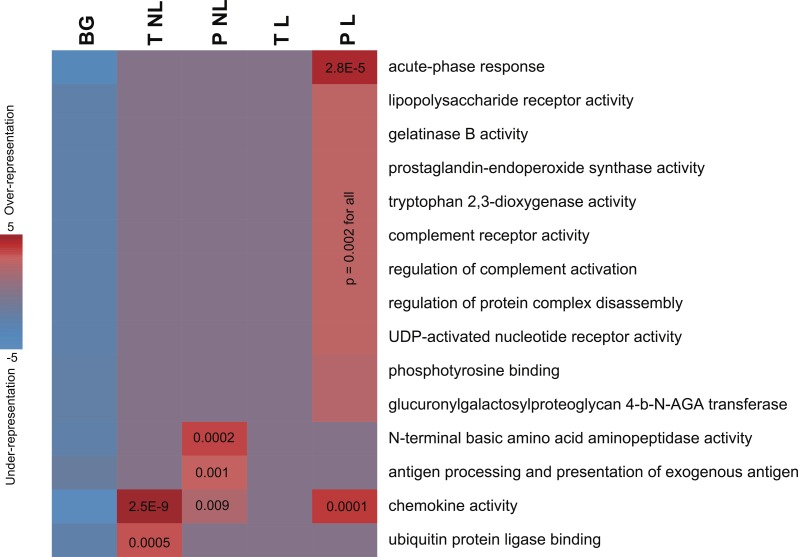
Informative pathways and their patterns of overrepresentation across the four phenotypes. In this representation, columns represent the four phenotypes of term non-labor (TNL), preterm non-labor (PNL), term labor (TL) and preterm labor (PL) as well as background (BG) gene expression pattern from published gene expression experiments in different tissues. Rows correspond to significantly informative pathways. Red indicates overrepresentation of pathway genes in a given phenotype, blue indicates underrepresentation, as measured using hypergeometric *p* values (log-transformed) and represented by color intensity.

#### Molecular pathways

The sets of transcripts identifying each of the four phenotypes showed significant enrichment in genes involved in infection, inflammation and immune response. No pathways were depleted or represented significantly less frequently than by chance alone. Pathway enrichment measures, assessed using hypergeometric *p* values after accounting for multiple comparisons, were highly significant (Fig. 2). PL was identified not only by the largest number of genes but also was associated with the largest number of unique pathways (Table 2), mainly associated with infection and inflammation. Conversely, among the genes identifying TL no pathways were over-represented. The sets of genes identifying TNL were significantly enriched in the chemokine genes and NFKBIA (Fig. 2). The genes identifying PNL belonged to antigen presentation pathways and anti-angiogenic chemokines (Fig. 2).

**Table 2:** Characteristics of the transcriptional profiles of the four phenotypes. Genes, number of genes which expression profile uniquely identifies one of the phenotypes in comparison to remaining three phenotypes. Pathways, number of pathways showing significant enrichment of genes which expression profile identifies each of the four phenotypes. Direction of the change in the transcription of genes identifying each phenotype, higher or lower, comparing to the other three phenotypes. In parenthesis number of genes with expression in opposite direction to the remaining genes identifying given phenotype.

Expression profile	Phenotype			
	P L	P NL	T L	T NL
Genes (*n*)	50	6	6	23
Pathways (*n*)	12	3	0	2
Direction of transcription change	HIGHER(5)	HIGHER	HIGHER (1)	LOWER

**Figure 3:**
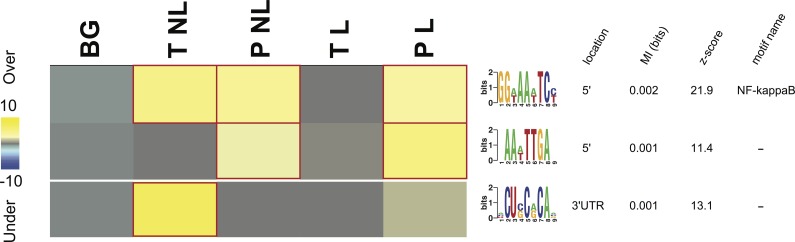
Overrepresentation patterns of the putative cis-regulatory elements across the four phenotypes. Rows correspond to the discovered putative regulatory elements and columns to the four phenotypes of term non-labor (TNL), preterm non-labor (PNL), term labor (TL) and preterm labor (PL) as well as background (BG) gene expression pattern from published gene expression experiments in different tissues. Yellow color indicates regulatory element overrepresentation in a given phenotype (measured by negative log-transformed hypergeometric *p* values) while blue color indicates underrepresentation (log-transformed hypergeometric *p* values). Significant over-representation (*p* < 0.05 after Bonferroni correction) is highlighted with red frames. Each sequence motif is shown 5′ to 3′ with relative nucleotide occurrence in the consensus (bits). For each motif, indicated is it’s: location in 5′ upstream region or 3′ UTR, mutual information (MI) value and *Z*-score associated with the MI value, corresponding to a number of SD from the mean and calculated with 10,000 randomization tests.

### Regulatory elements

#### Regulatory elements and labor

Three potential *cis*-regulatory sequences were identified in 5′- and 3′-UTR regions of the genes associated with the four phenotypes. Two of these sequences were located in 5′ promoter regions (motif A (GG[AT]AA[AT]TC[CT]) and B (AA[AT]TTGA)) and one (motif C ([ACU]CU[CG]C[AG]CA[ACG])) in 3′ UTR regions (Fig. 3). The motif A corresponds to the binding site for NF-kappa B (NFKB) and was significantly overrepresented in the promoter regions of the genes identifying PL, PNL and TNL (Fig. 3). The motif A association with the gene expression patterns of these three phenotypes was highly significant (*p* < 0.05 after Bonferroni correction and *Z* score associated with the mutual information value of 21.9, calculated with 10,000 randomization tests) and robust (robustness score of 9/10 obtained from ten jack-knife trials of randomly removing one-third of the genes and reassessing the statistical significance of the resulting mutual information values) (Fig. 3). The motif B did not correspond to any currently known transcription factors binding sites. It was significantly (*p* < 0.05 after Bonferroni correction and *Z* score associated with the mutual information value of 11.4) and robustly (robustness score of 6/10) overrepresented within 5′ promoter regions of the genes identifying PL and PNL (Fig. 3). The motif C also did not correspond to any currently known miRNA-targeting or regulatory enhancer sites, but it was highly informative about the expression profile of genes identifying TNL (Fig. 3). The motif C was significantly (*p* < 0.05 after Bonferroni correction and *Z* score of 13.1) and robustly (robustness score of 8/10) overrepresented in 3′ UTR regions of those genes (Fig. 3).

**Figure 4:**
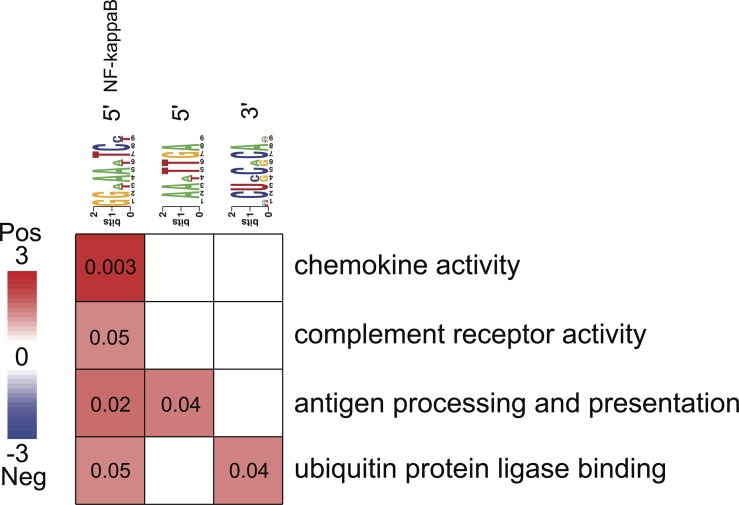
Associations between enriched pathways and cis-regulatory elements. Rows correspond to informative pathways and columns to informative regulatory elements. Red color indicates the degree of positive association in which genes belonging to a pathway are also enriched in a given regulatory element in the 5′ upstream promoter region or 3′ untranslated region (measured using log-transformed hypergeometric *p* values).

#### Regulatory elements and pathways

All three putative *cis*-regulatory elements were highly significantly associated with independently identified pathways (Fig. 4). Each motif and a corresponding pathway were mutually informative. Motif A, the NFKB binding site, was significantly over-represented in the 5′-UTR regions of genes belonging to chemokine activity, complement receptor activity, antigen processing and presentation and ubiquitin protein ligase binding pathways. The latter represented by nuclear factor of kappa light polypeptide gene enhancer in B-cells inhibitor, alpha (NFKBIA) gene (Fig. 4). The presence of motif B was significantly enriched in 5′-UTR regions of genes belonging to the antigen processing and presentation pathway (Fig. 4). The presence of motif C was enriched among the 3′-UTR regions of genes belonging to ubiquitin protein ligase binding pathway (Fig. 4).

#### Regulatory elements interactions

**Figure 5:**
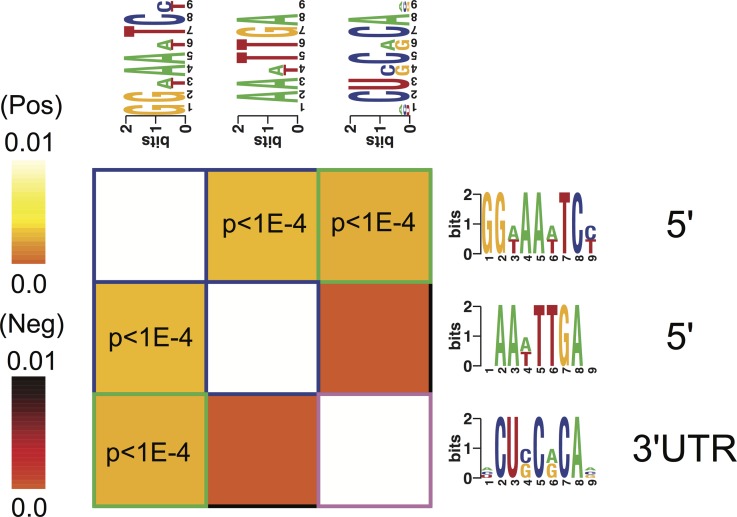
Cis-regulatory elements interactions. An interaction matrix is shown for cis-regulatory elements discovered among the genes identifying each of the four phenotypes. Rows and columns correspond to putative regulatory elements. The light colors white and yellow indicate significant motif co-occurrences. Presence of one regulatory element implies the presence (light color) or the absence (dark color) of another regulatory element within the promoter or 3′ UTR of the same gene. Statistically significant information values (*p* < 10–4) that involve homotypic regulatory elements pairs are highlighted with blue (5′–5′) while heterotypic pairs (5′–3′ UTR) are highlighted with green frames.

Motif A co-occurred with motifs B and C within respectively 5′- and 3′-UTR regulatory regions of the genes identifying the four phenotypes (Fig. 5). There was no significant correlation of the occurrence between motifs B and C (Fig. 5).

### PCR validation

**Figure 6:**
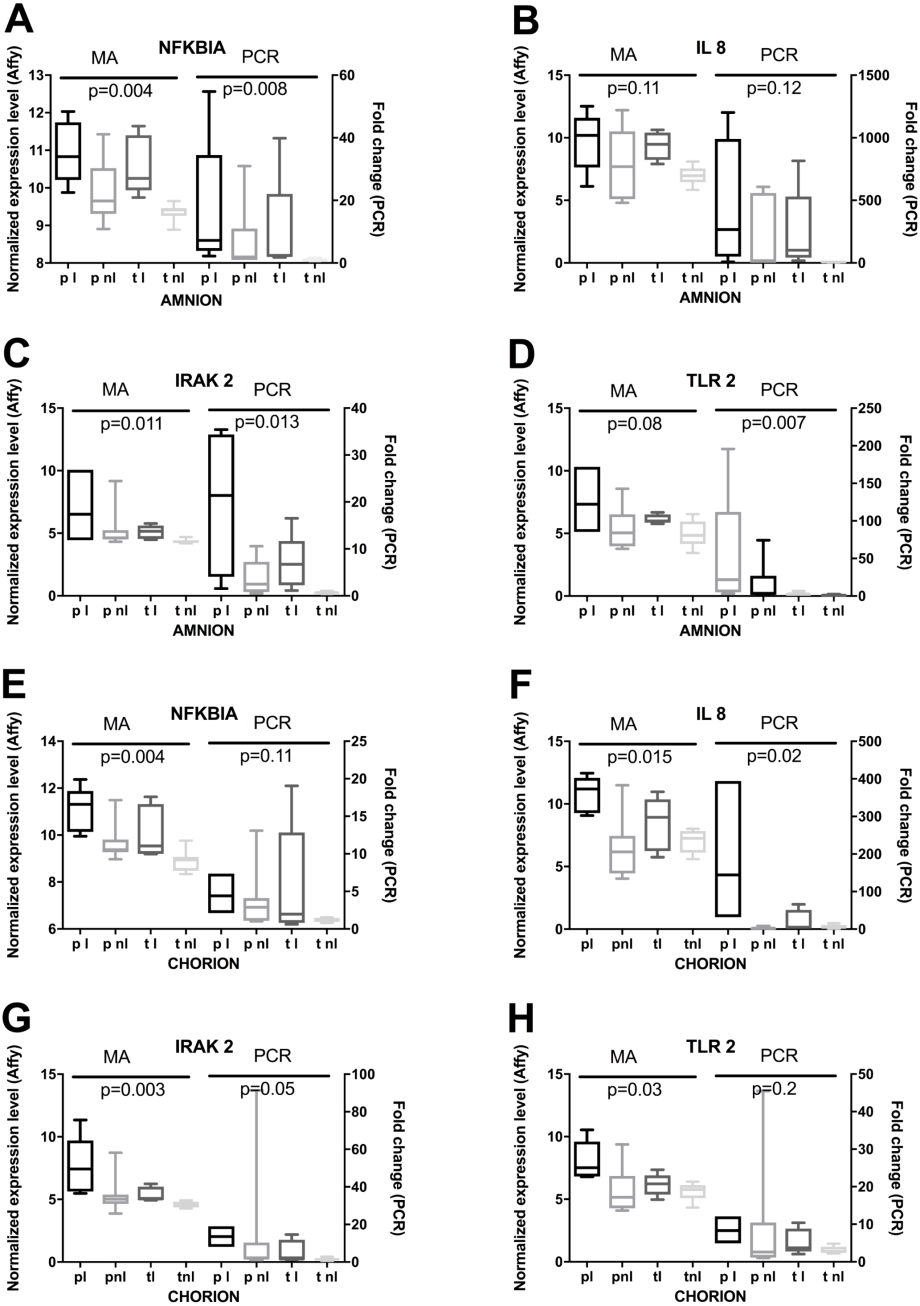
RT-qPCR based validation of expression changes, assessed by microarrays. (A) Amnion RNA for four genes was assayed by RT-qPCR (PCR) and the results were compared to microarray data (MA). Phenotypic groups (*x*-axis) are labeled as in Fig. 2, and *p*-values are shown from multi-class ANOVA testing the likelihood of difference across phenotypic groups. Boxes represent the 10th and 90th percentiles of distribution, with horizontal bars at the average and whiskers for the full range. (B) RT-qPCR and microarray results are shown as in (A) for RNA extracted from chorion.

**Figure 7:**
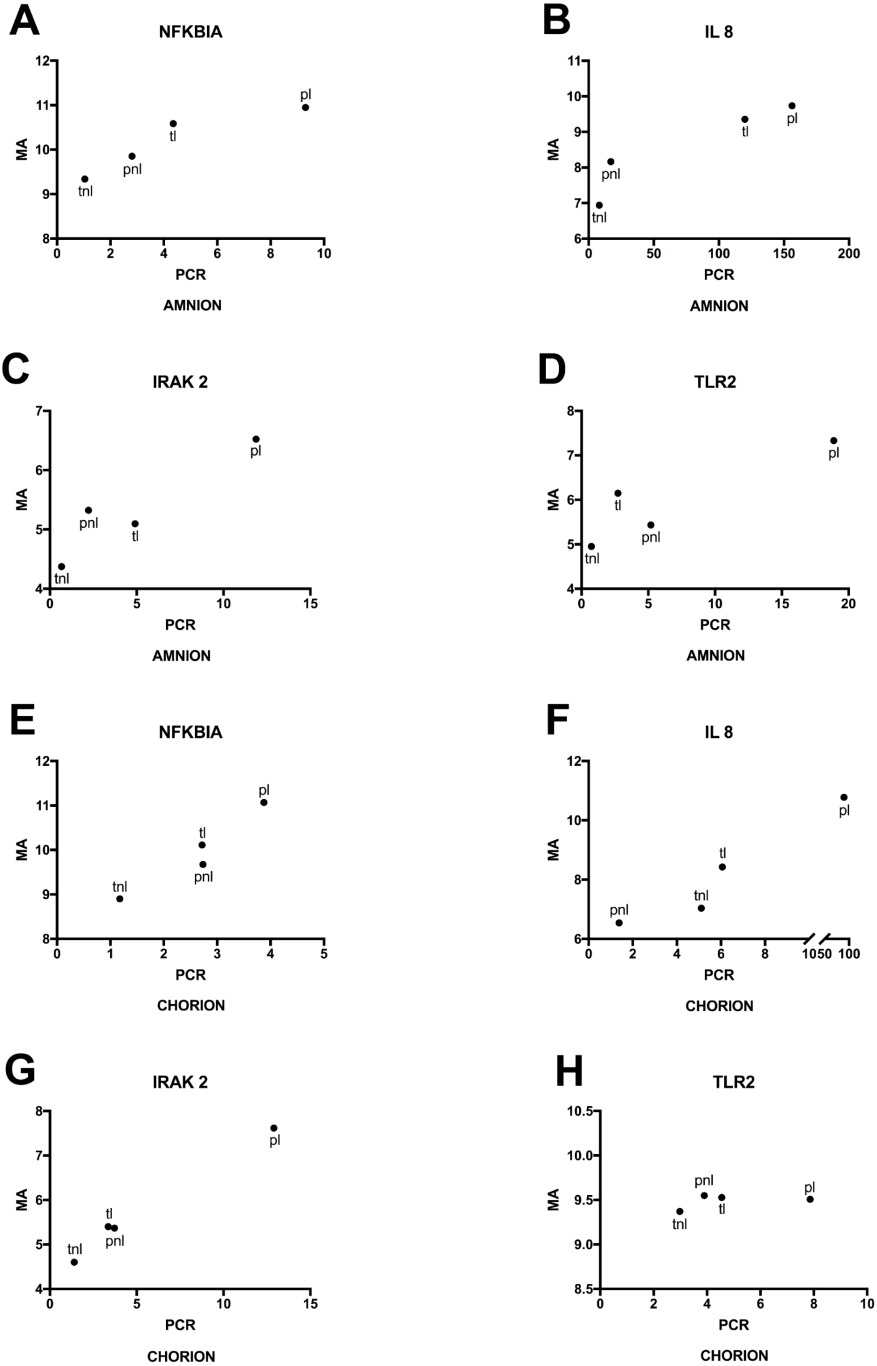
Mean gene expressions obtained using microarray and PCR in four phenotypes. (A) Amnion: Mean RNA expression for four genes assayed by RT-qPCR (PCR) and the microarray (MA). Phenotypic groups are labeled as in Fig. 6, (B) Chorion: RT-qPCR and microarray results are shown as in (A) for RNA extracted from chorion.

Lastly, we used RT-qPCR to validate mRNA expression profiles that were observed using microarrays. Selected candidates showed similar patterns among the four phenotypes for normalized expression levels and fold changes (Fig. 6). Significant differences in microarray assays among the four phenotypes were reproduced by RT-qPCR. The levels of expression of the four genes in the validation study showed significant differences among the four phenotypes either in the amnion or in the chorion (Fig. 6) and similar patterns of gene expression (Fig. 7).

## Discussion

### Findings

We have developed a model of the pregnancy gene expression profile to identify molecular processes underlying preterm and term labor and used the centroid based classification modeling to disentangle the molecular effects of two underlying processes of labor and prematurity. Our findings provide a novel insight into preterm and term parturition at the transcriptomic level: (1) They identify processes preceding and leading to the common final pathway of parturition, of uterine contractions, cervical changes and rupture of fetal membranes; (2) The findings show that the processes leading to the common final pathway of parturition are predominantly associated with regulation of immunological processes occurring at the maternal fetal interface; (3) Our data suggest that maintenance of pregnancy is associated with suppression of chemokines and cytokines, onset of labor at term is accompanied by withdrawal of this suppression and preterm labor is associated with overwhelming of the suppression by activation of the immune system. Placental impairment requiring preterm delivery of the fetus is associated with expression patterns of immune rejection.

The RNA gene expression characterizes very well pregnancy at term (86% of T NL samples), about a half of preterm labors (47% of P L samples), and poorly preterm pregnancies delivered for maternal or fetal indications (16% of P NL samples) or labor occurring at term (11% of T L samples) (Table 1).

These findings provide a fundamental insight into the processes of preterm and term labor which are congruent with prior research findings in the area. The expression profiles of the four phenotypes provide complementary insights and together a comprehensive image of parturition.

First, the expression of genes associated with maintenance of pregnancy (T NL) contribute a dominant part of gene expression profile of each phenotype. Gene expression profile of each phenotype uniquely characterizes this phenotype. However, since the overarching aim of each pregnancy is its maintenance, the gene expression profile associated with maintenance of pregnancy (T NL) together with the unique gene expression profile of a given phenotype could be seen as constituting the expression signature of each phenotype. Thus, in each phenotype samples classified as T NL expressing profile typical of maintenance of pregnancy may be considered as correctly classified and more completely describing the phenotype. Together with genes uniquely associated with a given phenotype, the genes associated with maintenance of pregnancy identify 91% of P L samples (47% P L and 44% T NL), 78% of P NL (16% P NL and 62% T NL), 61% of T L (11% T L and 50% T NL), and 86% of T NL samples (Table 1).

Second, it is also consistent with other knowledge that P NL phenotypes are associated additionally with P L expression profile. Many indications for preterm delivery of the baby in P NL (placenta previa, placental abruption or preterm premature rupture of the membranes with signs of infection) are known to initiate the preterm labor cascade (Norwitz, Robinson & Challis, 1999). Since 30% of the patients with P NL phenotype had those indications for delivery, this likely explains why 20% of P NL phenotypes have expression profile of P L.

Third, the observed heterogeneity of the expression profiles of T L phenotype is also plausible. T L specific expression profile has only six genes and no identifiable pathways (Table 2) indicating release of the uniform expression profile of chemokine suppression characterizing maintenance of pregnancy and return to baseline gene expression, a background gene expression pattern from published gene expression experiments in different tissues (Fig. 2).

### Mechanistic insights

Before onset of labor, normal term pregnancy is characterized by suppression of chemokines and cytokines, rendering it undetectable to the immune system and preventing influx of maternal immune cells necessary for onset of labor (Challis et al., 2009; Shynlova et al., 2013), but with the inflammatory pathway regulated by NFkB poised for activation. At term with onset of labor, withdrawal of the chemokine suppression leads to the term labor and birth, while preterm activation of the immune system overrides the chemokine suppression and results in preterm birth. The pregnancies delivered prematurely due to placental impairment disorders, such as intrauterine growth restriction, fetal distress, preeclampsia, placenta previa and placental abruption, showed gene expression patterns characteristic of immune rejection.

Preterm and term parturition involve a set of molecular processes associated with the uterine contractions, cervical changes and activation and rupture of fetal membranes, that are necessary for the delivery of the fetus, and are considered a common final pathway of labor (Norwitz, Robinson & Challis, 1999; Romero, Dey & Fisher, 2014). By considering four phenotypes (PL, PNL, TL, TNL) rather than only two phenotypes (e.g., PNL and PL) and using centroid based analysis, we identified gene expression signatures which differentiate each phenotype from the remaining three phenotypes (Tibshirani et al., 2002). This approach excluded expression changes shared by phenotypes, for example PL and TL, and associated with the final common pathway of labor. This in turn allowed the identification of molecular processes preceding and leading to the common final pathway of labor. These processes almost exclusively involve regulation of the immune system, and occurred predominantly at the maternal fetal interface, mainly in the decidua, chorion and amnion.

The gene expression profile of TNL represents the molecular processes underlying the maintenance of pregnancy. This expression profile shows that pregnancy is associated with local suppression of chemokines expression with simultaneous suppression of the inhibitors of the NFkB inflammatory pathway. Such chemokine suppression prevents chemotaxis of immune cells, such as effector T cells, from trafficking into gestational tissues such as the decidua, thus preventing the onset of labor (Matzinger & Kamala, 2011; Nancy & Erlebacher, 2014; Nancy et al., 2012). Simultaneous readiness of the NFkB inflammatory pathway suggests that pregnancy is poised to activate inflammatory cascades necessary for initiation of labor in preterm as well as in term birth (Challis et al., 2009; Christiaens et al., 2008; Osman et al., 2003; Romero, Dey & Fisher, 2014; Young et al., 2002). This observation is supported by animal experiments showing impaired accumulation of T-cells at the maternal fetal interface during pregnancy before onset of labor, partially due to epigenetic silencing of the chemokine genes in the decidua (Nancy et al., 2012). The pattern of expression characterizing term labor suggests withdrawal of the local chemokine suppression at the maternal fetal interface. The withdrawal of suppression and restoration of chemokines expression to normal levels may render the pregnancy detectable to components of the maternal immune system. The consequent influx of adaptive immune cells activates the final common contractile pathway and results in term birth (Challis et al., 2009; Christiaens et al., 2008; Osman et al., 2003; Romero, Dey & Fisher, 2014; Young et al., 2002). In preterm birth, the gene expression pattern suggests that a protective local chemokine suppression mechanism that contributes to the maintenance of pregnancy is overcome by activation of the multiple immune pathways. This may result in influx of immune cells and activation of the final common pathway and preterm birth. The induction of a pro-inflammatory response by the immune system is a well-recognized feature of the preterm labor (Christiaens et al., 2008; Romero, Dey & Fisher, 2014). Inflammation at the maternal-fetal interface is also apparent in conditions affecting placental function without evidence of labor, such as intrauterine growth restriction, fetal distress, preeclampsia, placenta previa or placental abruption (Chaiworapongsa et al., 2014; Challis et al., 2009; Madan et al., 2010; Nath et al., 2007; Redman & Sargent, 2005; Salafia et al., 1995a; Salafia et al., 1995b). Our data highlight a unique observation, that the PNL phenotype, in which preterm delivery is indicated without evidence of labor due to the conditions affecting placental function, is characterized by gene expression patterns characteristic of immune rejection (Belperio et al., 2003; Kitko et al., 2014; Wen et al., 2013) and inhibition of angiogenesis (Romagnani et al., 2004). Activation of the immune rejection pathways could result in maternal fetal interface impairment, which may indicate preterm delivery without spontaneous onset of labor (Aktas et al., 2014; Moffett & Colucci, 2014; Salafia et al., 1995a; Salafia et al., 1995b).

The gene expression profiles of the subset of tissues with the largest gene expression variability across the phenotypes decidua, chorion, amnion, and placenta classified well P L and T NL, but very poorly P NL and T L. This pattern may suggest that gene expression in those tissues at the maternal-fetal interface is important to the processes underlying preterm labor (P L) and maintenance of pregnancy (T NL). However, differences due to chance cannot be excluded.

The locations of putative regulatory elements in genes composing the expression signatures support insights into preterm and term birth and control of the immune system. Gene expression patterns associated with the maintenance of pregnancy (TNL phenotype) are involved in chemokine activity and show overrepresentation of the putative 5′-UTR regulatory elements corresponding to the binding site of NFKB and of a novel putative 3′-UTR regulatory element. The NFKB binding site is overrepresented in 5′-UTR of genes in both pathways associated with TNL, with chemokine activity and NFKB inhibitor A (NFKBIA) (ubiquitin protein ligase binding pathway), while the putative regulatory element is overrepresented only in the 3′-UTR of the latter. This observation suggests a basis for a negative feedback preventing chronic and escalating inflammation during pregnancy and maintenance of the pregnancy. NFKB is able to bind to the 5′-UTR region of NFKBIA and by inducing its transcription limit its own activation (Pahl, 1999). Such a negative feedback mechanism would result in the low expression of chemokines at the maternal fetal interface during pregnancy observed in this study. On the other hand, the genes whose expression patterns are associated with pathologic conditions of PL and PNL do not include NFKBIA and overrepresentation of the novel putative regulatory element in the 3′-UTR. Thus, the gene expression profiles in those conditions would lack the necessary element for the negative feedback mechanism and would show characteristics of chronic escalating inflammatory process, the findings observed in this study. However, the 5′-UTRs of genes whose expression pattern is associated with PL and PNL have overrepresentation of NFKB binding site and a putative novel regulatory element. Those co-occurring elements would allow activation of chemokines and complement observed in PL (Lindstrom & Bennett, 2005; McElroy et al., 2013). The presence of the NFKB biding site in relevant promoter regions provides a potential mechanism for activation of NFKB regulated inflammatory pathways, when chemokine suppression during pregnancy is overridden by immune system activation in preterm labor (Lim et al., 2012). Genes whose expression patterns characterize PNL are associated with the antigen processing and presentation and anti-angiogenic chemokine activity, pathways important in the process of immune rejection (Belperio et al., 2003; Wen et al., 2013). Overrepresentation in their 5′-UTR of NFKB binding site will also provide a mechanism for NFKB mediated immune rejection (Lee & Burckart, 1998).

### Strengths

The major strength of this study is a very precise phenotyping with standardized definitions of labor, including number of contractions and cervical change parameters, strict and extensive eligibility criteria, with inclusion of only preterm births at or before 34 weeks. The large gestational age difference in definitions of preterm and term births prevented misclassification of some term pregnancies as preterm due to uncertain gestational age estimates observed even in well-dated pregnancies, (Kierans et al., 2007).

Analysis of four phenotypes (PL, PNL, TL, TNL) rather than two, using the centroid based approach, allowed us to identify gene expression patterns which uniquely characterize each phenotype (Tibshirani et al., 2002). Such an approach eliminates transcripts that are associated with two or more phenotypes (e.g., PL and TL), thus eliminating from the analysis transcripts involved in the common final pathway of labor (Norwitz, Robinson & Challis, 1999; Romero, Dey & Fisher, 2014) and related to contraction, cervical change and membrane rupture which are common to the labor-associated phenotypes.

Rather than focusing on assessment of gene expression among different phenotypes in a single tissue, we compared gene expression profiles of *all* tissues comprising the pregnant uterus in the same individual. Whereas prior studies have shown that gene expression changes in many tissues are associated with term and preterm labor (Bukowski et al., 2006; Chaemsaithong et al., 2013; Chan et al., 2002; Chim et al., 2012; Esplin et al., 2005a; Esplin et al., 2005b; Hassan et al., 2006; Hassan et al., 2007; Hassan et al., 2010; Havelock et al., 2005; Heng et al., 2014; Lim et al., 2012; Mittal et al., 2011; Mittal et al., 2010; Nhan-Chang et al., 2010; Romero et al., 2014) our approach enabled us to define the decidua, chorion and amnion as sites most relevant to initiation of parturition signals.

In the pregnant uterus, different tissues are in close proximity and molecular signals easily affect adjacent tissues in a paracrine fashion (Norwitz, Robinson & Challis, 1999; Racicot et al., 2014; Romero, Dey & Fisher, 2014; Smith, 2007). This renders the pregnant uterus a well-coordinated, functional unit. Simultaneous evaluation of all transcripts in the uterine tissues enhanced our ability to detect differences and decreased the probability of false positive findings due to spurious findings in one tissue.

The approach applied in this study of 10 times 10 fold cross-validation and shrinkage in centroid analysis decreases overfitting, a main limitation of high throughput methods, which occurs when the data contain large number of predictors—genes in comparison to number of samples, subjects and outcomes (Everitt & Skrondal, 2010). Both elements of the analytic approach used in this study, the shrinkage in centroid analysis and 10 times 10-fold cross-validation, were shown to minimize overfitting and thus to improve generalizability of the findings (Shi et al., 2010; Steyerberg et al., 2001). In addition to the cross-validation, we validated our results by RT-qPCR, and by providing the biological plausibility of the associated molecular pathways and independently, the putative regulatory elements binding sites.

### Limitations

Unequal size of the phenotypic groups in the analysis is a limitation of this study. The unequal size of the groups poses a risk of class imbalance problem, where the classifier assigns most of the new samples to the largest class or group to minimize the prediction error and results in poor predictive accuracy for the smaller classes or groups (Blagus & Lusa, 2013). The class imbalance bias unlikely had a major impact on this study’s findings, because although majority of samples were assigned to the largest group T NL, the second most often predicted class P L was the smallest of the four groups. Moreover, opposite to what would be expected in class imbalance bias, the predictive accuracy for the smallest groups T L and P L was actually higher than for the larger groups.

Due to a relatively small number of subjects in each phenotype, the study has insufficient power to detect gene expression differences among the phenotypes in each tissue separately. Thus, differences in transcript expression among the phenotypes that were very similar in magnitude but in opposite directions in different tissues would not be detected using our approach. Such constellation of gene expression patterns in different tissues is unlikely and could potentially apply to very few genes. Moreover, if present, it would result in false negative findings and thus would not affect the genes expression patterns reported in this study.

A second limitation is lack of data on gene expression patterns in the uterine cervix due to insufficient number of specimens. However, since structural cervical changes are a part of the common final pathway of labor it is unlikely that its gene expression profile would contribute significantly to the molecular processes preceding and leading to the common final pathway of labor. The gene expression changes of the cervix in the second trimester associated with cervical shortening predicting preterm birth or cervical insufficiency would also not be detected using this study design. However, those conditions are respectively not associated with the process of labor itself but rather predict it or are associated with different than preterm labor condition.

The cross-validation used to determine the amount of shrinkage was separate and was not nested within the cross-validation used to determine the classification performance. The cross-validation loops used to determine the amount of shrinkage and the classification performance were separate and were not nested. This approach is associated with a risk of over-fitting, especially in non-linear models with a large number of parameters. However, in a model with a single parameter, shrinkage, the risk of over-fitting is likely small. We re-analyzed the data using 10×-replicated 10-fold cross-validation using an additional 10-fold inner nested cross-validation within each fold to select the shrinkage parameter with the essentially unchanged classification accuracy of the nested and non-nested approach (overall estimated accuracy of 40.98% and 42.02%, respectively). The proportion of correctly classified individual phenotypes was also very similar using nested and non-nested approaches (PL 41% vs. 47%; PNL 18% vs. 16%; TL 11% vs. 11%; TNL 84% vs. 86%, respectively).

Finally, the PNL phenotype group is relatively small thus subject to dominance by the characteristics of the most prevalent etiologies e.g., preeclampsia and fetal growth restriction. Therefore, interpretation of the gene expression profile associated with this phenotype needs to account for these limitations.

### Summary

In conclusion, the findings of this study show that pregnancy is maintained by downregulation of chemokines at the maternal fetal interface preventing influx of immune cells and thus onset of labor. The chemokine suppression during pregnancy is withdrawn at the time of term birth and overridden by the activation of multiple pathways of the immune system in the preterm birth (Fig. S1). Complications of the pregnancy associated with impairment of placental function, intrauterine growth restriction, preeclampsia, fetal distress, placenta previa and placental abruption, necessitating preterm delivery of the fetus in absence of labor, show gene expression patterns associated with immune rejection.

##  Supplemental Information

10.7717/peerj.3685/supp-1 Figure S1 A model of regulation of the inflammatory process associated with onset of preterm and term labor

10.7717/peerj.3685/supp-2 Figure S2 Shrunken differences in the RNA expression of the genes uniquely identifying the four phenotypes Shrunken expression differences are nearly mutually exclusive.

10.7717/peerj.3685/supp-3 Table S1 Genes identifying preterm labor Genes in bold font were expressed higher in this group than in the other three groups. Genes not in bold font were expressed lower in this group than in the other three groups.

10.7717/peerj.3685/supp-4 Table S2 Genes identifying preterm non-labor All gene products were expressed higher in this group than in the other three groups.

10.7717/peerj.3685/supp-5 Table S3 Genes identifying term labor Genes in bold font were expressed higher in this group than in the other three groups. Genes not in bold font were expressed lower in this group than in the other three groups.

10.7717/peerj.3685/supp-6 Table S4 Genes identifying term non-labor All gene products were expressed lower in this group than in the other three groups.

10.7717/peerj.3685/supp-7 Table S5 Primers used in the qPCR validation

## Additional Information and Declarations

### Data Availability

The following information was supplied regarding data availability: Microarray data are available at the Gene Expression Omnibus under accession number GSE73685.
