# Supplementary figures and images for: Onset of human preterm and term birth is related to unique inflammatory transcriptome profiles at the maternal fetal interface
Source: PeerJ. 2017 Sep 1;5:e3685. doi: 10.7717/peerj.3685 (PMC5582610; doi:10.7717/peerj.3685)

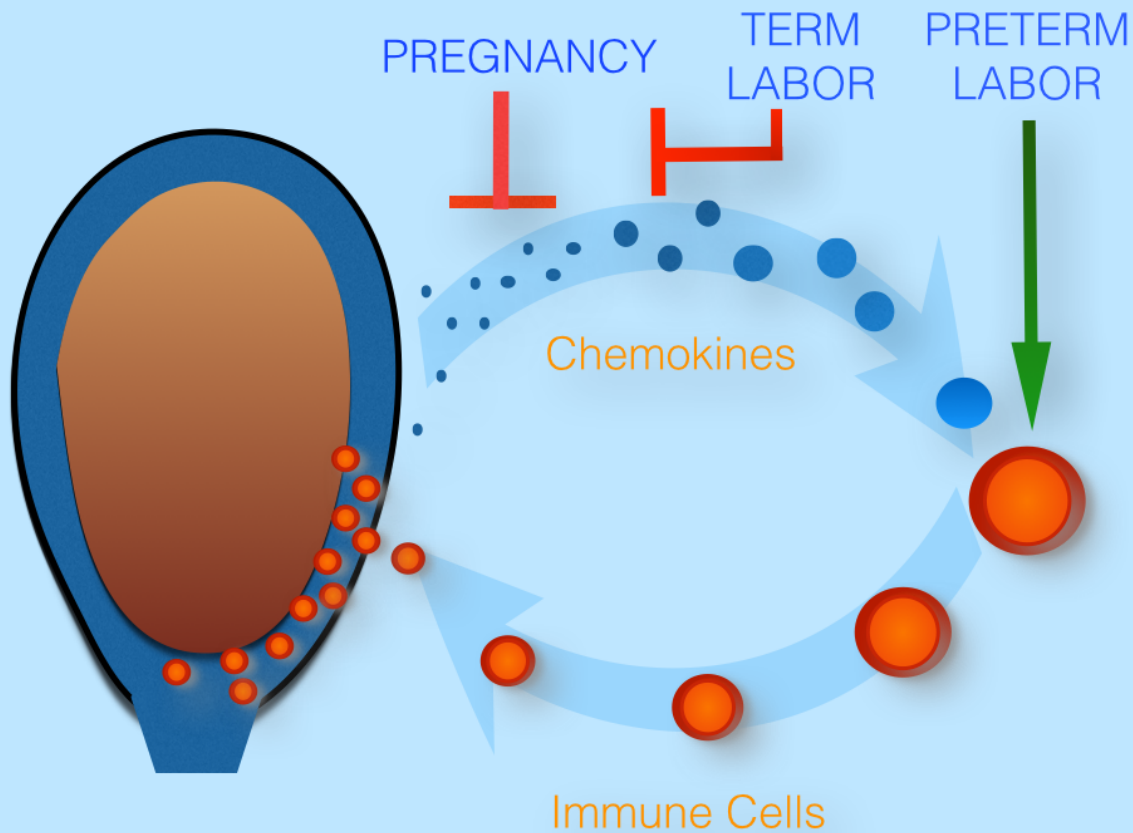

Supplement: Figure S1 [file peerj-05-3685-s001.pdf]
