# Supplementary material for: Onset of human preterm and term birth is related to unique inflammatory transcriptome profiles at the maternal fetal interface
Source: PeerJ. 2017 Sep 1;5:e3685. doi: 10.7717/peerj.3685 (PMC5582610; doi:10.7717/peerj.3685)
Supplement: Figure S2 — Shrunken expression differences are nearly mutually exclusive. [file peerj-05-3685-s002.pdf]

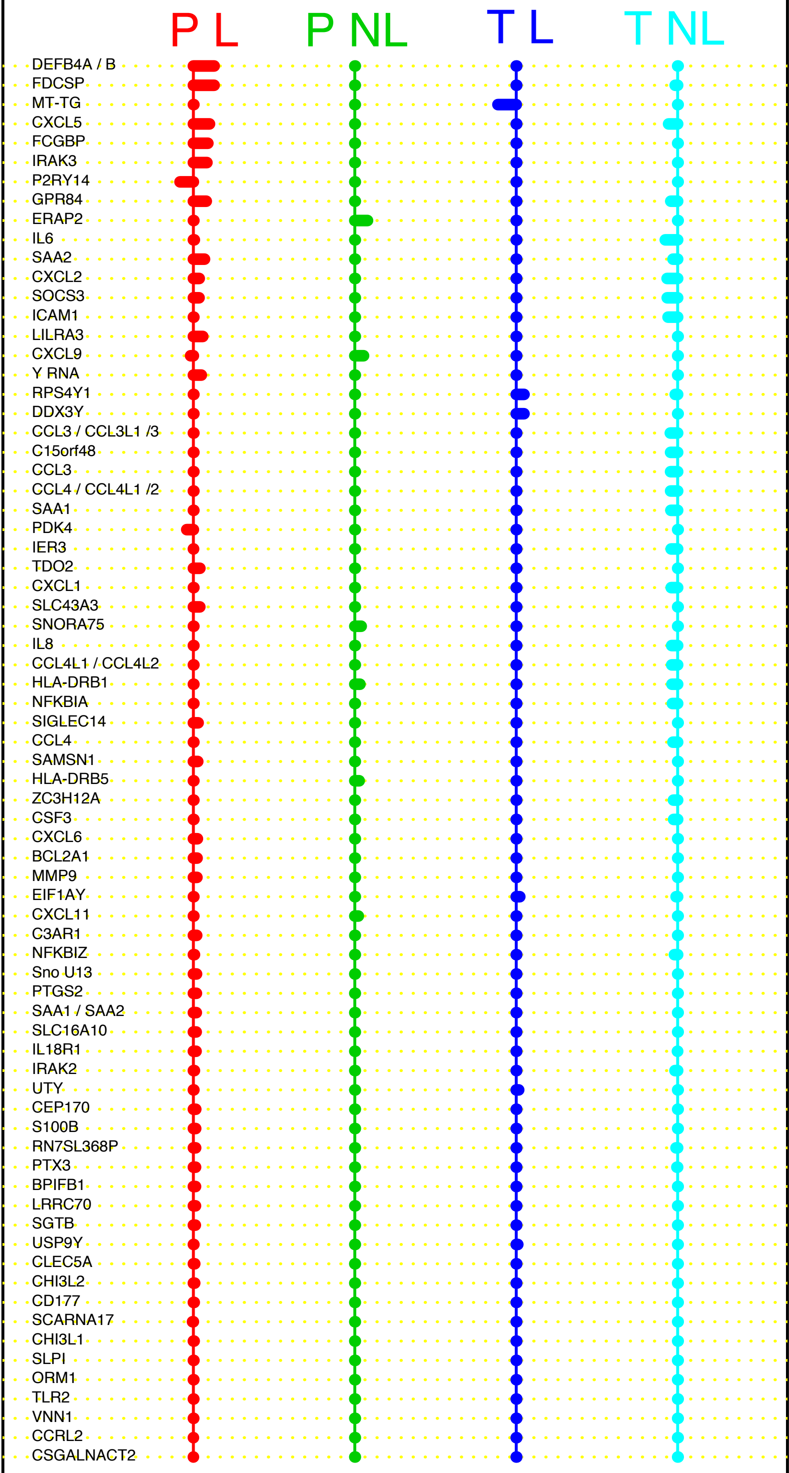

P L
P NL
T L
T NL
DEFB4A / B
FDCSP
MT-TG
CXCL5
FCGBP
IRAK3
P2RY14
GPR84
ERAP2
IL6
SAA2
CXCL2
SOCS3
ICAM1
LILRA3
CXCL9
Y RNA
RPS4Y1
DDX3Y
CCL3 / CCL3L1 /3
C15orf48
CCL3
CCL4 / CCL4L1 /2
SAA1
PDK4
IER3
TDO2
CXCL1
SLC43A3
SNORA75
IL8
CCL4L1 / CCL4L2
HLA-DRB1
NFKBIA
SIGLEC14
CCL4
SAMSN1
HLA-DRB5
ZC3H12A
CSF3
CXCL6
BCL2A1
MMP9
EIF1AY
CXCL11
C3AR1
NFKBIZ
Sno U13
PTGS2
SAA1 / SAA2
SLC16A10
IL18R1
IRAK2
UTY
CEP170
S100B
RN7SL368P
PTX3
BPIFB1
LRRC70
SGTB
USP9Y
CLEC5A
CHI3L2
CD177
SCARNA17
CHI3L1
SLPI
ORM1
TLR2
VNN1
CCRL2
CSGALNACT2
