# Supplementary material for: Onset of human preterm and term birth is related to unique inflammatory transcriptome profiles at the maternal fetal interface
Source: PeerJ. 2017 Sep 1;5:e3685. doi: 10.7717/peerj.3685 (PMC5582610; doi:10.7717/peerj.3685)
Supplement: Table S1 — Genes in bold font were expressed higher in this group than in the other three groups. Genes not in bold font were expressed lower in this group than in the other three groups. [file peerj-05-3685-s003.docx]

| **Probe ID** | **Gene symbol** | **Gene name** |
| --- | --- | --- |
| **Toll-like receptor signaling and regulation** | | |
| **8105579** | **LRRC70** | **leucine rich repeat containing 70** |
| **8083594** | **PTX3** | **pentraxin 3, long** |
| **8061894** | **BPIFB1** | **BPI fold containing family B, member 1** |
| **7956878** | **IRAK3** | **interleukin-1 receptor-associated kinase 3** |
| **8077786** | **IRAK2** | **interleukin-1 receptor-associated kinase 2** |
| **8018864** | **SOCS3** | **suppressor of cytokine signaling 3** |
| **8081386** | **NFKBIZ** | **nuclear factor of kappa light polypeptide gene enhancer in B-cells inhibitor, zeta** |
| **8097903** | **TLR2** | **toll-like receptor 2** |
| **Cytokines, chemokines and their receptors** | | |
| **8079407** | **CCRL2** | **chemokine (C-C motif) receptor-like 2** |
| **8100994** | **CXCL2** | **chemokine (C-X-C motif) ligand 2** |
| **8100977** | **CXCL5** | **chemokine (C-X-C motif) ligand 5** |
| **8095688** | **CXCL6** | **chemokine (C-X-C motif) ligand 6 (granulocyte chemotactic protein 2)** |
| 8101118 | CXCL9 | chemokine (C-X-C motif) ligand 9 |
| **8131803** | **IL6** | **interleukin 6 (interferon, beta 2)** |
| **8044035** | **IL18R1** | **interleukin 18 receptor 1** |
| 8141094 | PDK4 | pyruvate dehydrogenase kinase, isozyme 4 |
| **Other immune & inflammation pathways** | | |
| **8143471** | **CLEC5A** | **C-type lectin domain family 5, member A** |
| **8129618** | **VNN1** | **vanin 1** |
| **8095467** | **FDCSP** | **follicular dendritic cell secreted protein** |
| **8038885** | **SIGLEC14** | **sialic acid binding Ig-like lectin 14** |
| **7960874** | **C3AR1** | **complement component 3a receptor 1** |
| **7923547** | **CHI3L1** | **chitinase 3-like 1 (cartilage glycoprotein-39)** |
| **7938758** | **SAA1** | **serum amyloid A1** |
| **7946983** | **SAA2** | **serum amyloid A2** |
| **7946986** | **SAA1 / SAA2** | **serum amyloid A1 and A2** |
| **8157446** | **ORM1** | **orosomucoid 1** |
| **8039226** | **LILRA3** | **leukocyte immunoglobulin-like receptor, subfamily A (without TM domain), member 3** |
| **7963770** | **GPR84** | **G protein-coupled receptor 84** |
| **Anti-microbial factors** | | |
| **8066493** | **SLPI** | **secretory leukocyte peptidase inhibitor** |
| **8144481** | **DEFB4A / B** | **defensin, beta 4A and 4B** |
| **Factors associated with antibody production and function** | | |
| **8036787** | **FCGBP** | **Fc fragment of IgG binding protein** |
| **Apoptosis pathways and regulation** | | |
| **7990818** | **BCL2A1** | **BCL2-related protein A1** |
| **8124848** | **IER3** | **immediate early response 3** |
| **Genes associated with fetal membrane rupture and uterine contractions** | | |
| **7922976** | **PTGS2** | **prostaglandin-endoperoxide synthase 2 (prostaglandin G/H synthase and cyclooxygenase)** |
| **8063115** | **MMP9** | **matrix metallopeptidase 9 (gelatinase B, 92kDa gelatinase, 92kDa type IV collagenase)** |
| **Various pathways** | | |
| **7903920** | **CHI3L2** | **chitinase 3-like 2** |
| **7927146** | **CSGALNACT2** | **chondroitin sulfate N-acetylgalactosaminyltransferase 2** |
| **7948229** | **SLC43A3** | **solute carrier family 43, member 3** |
| **8121515** | **SLC16A10** | **solute carrier family 16, member 10 (aromatic amino acid transporter)** |
| **7988438** | **Sno U13** | **Small nucleolar non-coding RNA U13** |
| 8021183 | SCARNA17 | small Cajal body-specific RNA 17 |
| **8037387** | **RN7SL368P** | **Non-coding cytoplasmic RNA** |
| **8155493** | **Y RNA** | **small non-coding RNA a component of the Ro60 a target of autoimmune antibodies** |
| **8037298** | **CD177** | **CD177 molecule** |
| **8069541** | **SAMSN1** | **SAM domain, SH3 domain and nuclear localization signals 1** |
| **8071036** | **S100B** | **S100 calcium binding protein B** |
| 8091511 | P2RY14 | purinergic receptor P2Y, G-protein coupled, 14 |
| **8112409** | **SGTB** | **small glutamine-rich tetratricopeptide repeat (TPR)-containing, beta** |
| **7925525** | **CEP170** | **centrosomal protein 170kDa** |
| **8097991** | **TDO2** | **tryptophan 2,3-dioxygenase** |
