# Supplementary material for: Onset of human preterm and term birth is related to unique inflammatory transcriptome profiles at the maternal fetal interface
Source: PeerJ. 2017 Sep 1;5:e3685. doi: 10.7717/peerj.3685 (PMC5582610; doi:10.7717/peerj.3685)
Supplement: Table S2 — All gene products were expressed higher in this group than in the other three groups. [file peerj-05-3685-s004.docx]

| **Probe ID** | **Gene symbol** | **Gene name** |
| --- | --- | --- |
| **Antigen Presentation** | | |
| **8107044** | **ERAP2** | **endoplasmic reticulum aminopeptidase 2** |
| **8125447** | **HLA-DRB1** | **major histocompatibility complex, class II, DR beta 1** |
| **8125436** | **HLA-DRB5** | **major histocompatibility complex, class II, DR beta 5** |
| **Cytokines, anti-angiogenic chemokines and their receptors** | | |
| **8101118** | **CXCL9** | **chemokine (C-X-C motif) ligand 9** |
| **8101131** | **CXCL11** | **chemokine (C-X-C motif) ligand 11** |
| **Various pathways** | | |
| **7961024** | **SNORA75** | **Small nucleolar RNA SNORA75** |
