# Supplementary material for: Onset of human preterm and term birth is related to unique inflammatory transcriptome profiles at the maternal fetal interface
Source: PeerJ. 2017 Sep 1;5:e3685. doi: 10.7717/peerj.3685 (PMC5582610; doi:10.7717/peerj.3685)
Supplement: Table S3 — Genes in bold font were expressed higher in this group than in the other three groups. Genes not in bold font were expressed lower in this group than in the other three groups. [file peerj-05-3685-s005.docx]

| **Probe ID** | **Gene symbol** | **Gene name** |
| --- | --- | --- |
| **Various pathways** | | |
| 8165672 | MT-TG | mitochondrially encoded tRNA glycine |
| **8176375** | **RPS4Y1** | **ribosomal protein S4, Y-linked 1** |
| **8176578** | **USP9Y** | **ubiquitin specific peptidase 9, Y-linked** |
| **8176624** | **DDX3Y** | **DEAD (Asp-Glu-Ala-Asp) box polypeptide 3, Y-linked** |
| **8176719** | **EIF1AY** | **eukaryotic translation initiation factor 1A, Y-linked** |
| **8177137** | **UTY** | **ubiquitously transcribed tetratricopeptide repeat gene, Y-linked** |
