# Supplementary material for: Onset of human preterm and term birth is related to unique inflammatory transcriptome profiles at the maternal fetal interface
Source: PeerJ. 2017 Sep 1;5:e3685. doi: 10.7717/peerj.3685 (PMC5582610; doi:10.7717/peerj.3685)
Supplement: Table S4 — All gene products were expressed lower in this group than in the other three groups. [file peerj-05-3685-s006.docx]

| **Probe ID** | **Gene symbol** | **Gene name** |
| --- | --- | --- |
| **Toll-like receptor signaling and regulation** | | |
| 8081386 | NFKBIZ | nuclear factor of kappa light polypeptide gene enhancer in B-cells inhibitor, zeta |
| 7978644 | NFKBIA | nuclear factor of kappa light polypeptide gene enhancer in B-cells inhibitor, alpha |
| 8018864 | SOCS3 | suppressor of cytokine signaling 3 |
| 8025601 | ICAM1 | intercellular adhesion molecule 1 |
| 8077786 | IRAK2 | interleukin-1 receptor-associated kinase 2 |
| **Cytokines, chemokines and their receptors** | | |
| 8131803 | IL6 | interleukin 6 (interferon, beta 2) |
| 8095680 | IL8 | interleukin 8 |
| 8095697 | CXCL1 | chemokine (C-X-C motif) ligand 1 (melanoma growth stimulating activity, alpha) |
| 8100994 | CXCL2 | chemokine (C-X-C motif) ligand 2 |
| 8100977 | CXCL5 | chemokine (C-X-C motif) ligand 5 |
| 8014391 | CCL3 / CCL3L1 /3 | chemokine (C-C motif) ligand 3; chemokine (C-C motif) ligand 3-like 1 and -like 3 |
| 8014369 | CCL3 | chemokine (C-C motif) ligand 3 |
| 8006602 | CCL4 | chemokine (C-C motif) ligand 4 |
| 8006608 | CCL4L1 / CCL4L2 | chemokine (C-C motif) ligand 4-like 1 and 4-like 2 |
| 8006621 | CCL4 / CCL4L1 /2 | chemokine (C-C motif) ligand 4; chemokine (C-C motif) ligand 4-like 1 and -like 2 |
| **Other immune & inflammation pathways** | | |
| 7900146 | ZC3H12A | zinc finger CCCH-type containing 12A |
| 7938758 | SAA1 | serum amyloid A1 |
| 7946983 | SAA2 | serum amyloid A2 |
| 7963770 | GPR84 | G protein-coupled receptor 84 |
| **Apoptosis pathways and regulation** | | |
| 8179704 | IER3 | immediate early response 3 |
| **Antigen Presentation** | | |
| 8125447 | HLA-DRB1 | major histocompatibility complex, class II, DR beta 1 |
| **Various pathways** | | |
| 8006999 | CSF3 | colony stimulating factor 3 (granulocyte) |
| 7983478 | C15orf48 | chromosome 15 open reading frame 48 |
