# Supplementary material for: Onset of human preterm and term birth is related to unique inflammatory transcriptome profiles at the maternal fetal interface
Source: PeerJ. 2017 Sep 1;5:e3685. doi: 10.7717/peerj.3685 (PMC5582610; doi:10.7717/peerj.3685)
Supplement: Table S5 [file peerj-05-3685-s007.docx]

| **Gene** | **Strain** | **Sequence** |
| --- | --- | --- |
| NFKBIA-F | F | CTCCATCCTGAAGGCTACCA |
| NFKBIA-R | R | GACACCAAAAGCTCCACGAT |
| IRAK2-F | F | AGAAGATGCCCCTCATTCCT |
| IRAK2-R | R | TCCAGCCAAGCTCAAAAGTT |
| IL8-F | F | CTGCGCCAACACAGAAATTA |
| IL8-R | R | ACTTCTCCACAACCCTCTGC |
| TLR2-F | F | GATGCCTACTGGGTGGAGAA |
| TLR2-R | R | CCACTTGCCAGGAATGAAGT |
| GAPDH-F | F | GAAGGTCGGAGTCAACGGATTT |
| GAPDH-R | R | GAATTTGCCATGGGTGGAAT |
